# A CCR5^+^ memory subset within HIV-1-infected primary resting CD4^+^ T cells is permissive for replication-competent, latently infected viruses in vitro

**DOI:** 10.1186/s13104-019-4281-5

**Published:** 2019-04-29

**Authors:** Kazutaka Terahara, Ryutaro Iwabuchi, Masahito Hosokawa, Yohei Nishikawa, Haruko Takeyama, Yoshimasa Takahashi, Yasuko Tsunetsugu-Yokota

**Affiliations:** 1 Department of Immunology, National Institute of Infectious Diseases, 1-23-1 Toyama, Shinjuku-ku, Tokyo, 162-8640 Japan; 2 Department of Life Science and Medical Bioscience, Waseda University, 2-2 Wakamatsu-cho, Shinjuku-ku, Tokyo, 162-8480 Japan; 3 Computational Bio Big-Data Open Innovation Laboratory, National Institute of Advanced Industrial Science and Technology, 3-4-1 Okubo, Shinjuku-ku, Tokyo, 169-8555 Japan; 4 Research Organization for Nano & Life Innovation, Waseda University, 513 Wasedatsurumaki-cho, Shinjuku-ku, Tokyo, 162-0041 Japan; 5 Institute for Advanced Research of Biosystem Dynamics, Waseda Research Institute for Science and Engineering, Waseda University, 2-2 Wakamatsu-cho, Shinjuku-ku, Tokyo, 162-8480 Japan; 6 Department of Medical Technology, School of Human Sciences, Tokyo University of Technology, 5-23-22 Nishikamata, Ota-ku, Tokyo, 144-8535 Japan

**Keywords:** HIV, Latent reservoir, Resting CD4^+^ T cells

## Abstract

**Objective:**

Resting CD4^+^ T cells are major reservoirs of latent HIV-1 infection, and may be formed during the early phase of the infection. Although CCR5-tropic (R5) HIV-1 is highly transmissible during the early phase, newly infected individuals have usually been exposed to a mixture of R5 and CXCR4-tropic (X4) viruses, and X4 viral DNA is also detectable in the host. Our aim was to identify which subsets of resting CD4^+^ T cells contribute to forming the latent reservoir in the presence of both X4 and R5 viruses.

**Results:**

Primary resting CD4^+^ naïve T (T_N_) cells, CCR5^−^ memory T (T_M_) cells, and CCR5^+^ T_M_ cells isolated by flow cytometry were infected simultaneously with X4 and R5 HIV-1, which harbored different reporter genes, and were cultured in the resting condition. Flow cytometry at 3 days post-infection demonstrated that X4 HIV-1^+^ cells were present in all three subsets of cells, whereas R5 HIV-1^+^ cells were present preferentially in CCR5^+^ T_M_ cells, but not in T_N_ cells. Following CD3/CD28-mediated activation at 3 days post-infection, numbers of R5 HIV-1^+^ cells and X4 HIV-1^+^ cells increased significantly only in the CCR5^+^ T_M_ subset, suggesting that it provides a major reservoir of replication-competent, latently infected viruses.

**Electronic supplementary material:**

The online version of this article (10.1186/s13104-019-4281-5) contains supplementary material, which is available to authorized users.

## Introduction

Current combination antiretroviral therapy has been successful in suppressing HIV-1 replication to undetectable levels. However, a barrier to the complete eradication of HIV-1 infection by combination antiretroviral therapy is the existence of a small reservoir of latently infected cells [1–4]. A prime candidate for this reservoir is resting CD4^+^ T cells since they are long-lived and can harbor replication-competent proviruses that remain transcriptionally silent in the absence of an activating stimulus [5–8]. Resting CD4^+^ T cells are heterogeneous populations that include naïve (T_N_) and memory (T_M_) cells. T_M_ cells are further divided into central memory (T_CM_), transitional memory (T_TM_), and effector memory (T_EM_) cells. Resting CD4^+^ T_M_ cells have been proposed to be major reservoirs of latent HIV-1 infection, on the evidence of high levels of HIV-1 DNA content [5, 9, 10]. However, it has also been suggested that resting CD4^+^ T_N_ cells are an important reservoir of latent HIV-1 infection [11, 12].

A latent reservoir could be established during the early phase of HIV-1 infection [1, 6], during which CCR5-tropic (R5) HIV-1 is highly transmissible [13–15]. Notably, results from next-generation sequencing suggest that CXCR4-tropic (X4) HIV-1 may be more prevalent during the early phase of HIV-1 infection than previously reported [16], so that newly infected individuals have usually been exposed to a mixture of X4 and R5 viruses [17–20]. CD4^+^ T cells undergoing effector-to-memory transition are permissive for HIV-1 latent infection [21]. Latency has also been shown to occur following direct infection of resting CD4^+^ T cells [22], but it is not yet known which subsets of resting CD4^+^ T cells are involved in the latent infection by X4 and R5 HIV-1.

We previously constructed a recombinant X4 HIV-1 (HIV-1_NL-E_) harboring EGFP reporter gene for expression of a green fluorescent protein, along with an isogenic R5 HIV-1 (HIV-1_NLAD8-D_) harboring DsRed gene, for expression of a red fluorescent protein, enabling us to distinguish between these viruses in productively infected cells [23]. Here, we investigated the infectivity of these viruses in isolated, primary human resting CD4^+^ T cell subsets (T_N_, CCR5^−^ T_M_, and CCR5^+^ T_M_) in a dual-infection model.

## Main text

### Methods

#### Virus preparation

To generate HIV-1_NL-E_ and HIV-1_NLAD8-D_ stocks, HEK293T cells were transfected with the corresponding proviral DNA plasmid using the calcium phosphate precipitation method as described previously [23]. The amount of p24 Gag in the culture supernatant was measured with an in-house enzyme-linked immunosorbent assay [24]. The supernatant was then filtered, aliquoted, and stored at − 80 °C.

#### Cell preparation

Human peripheral blood was donated by healthy Japanese adult volunteers. Peripheral blood mononuclear cells (PBMCs) were separated by the Lymphocyte Separation Medium 1077 (PromoCell, Heidelberg, Germany). CD4^+^ T cells were first negatively enriched from PBMCs using the EasySep Human CD4^+^ T cell Enrichment Kit (StemCell Technologies, Vancouver, BC, Canada). Enriched CD4^+^ T cells were stained with the following antibodies: CD69-FITC (FN50; ThermoFisher Scientific, Waltham, MA, USA), HLA-DR-Alexa Fluor 488 (L243; BioLegend, San Diego, CA, USA), CD8-PerCP (RPA-T8, BioLegend), CD19-PerCP (HIB19; BioLegend), CD27-Alexa Fluor 700 (O323; BioLegend), CD45RA-PE-Cy7 (HI100; BioLegend), and CCR5-Alexa Fluor 647 (T312; [25]). Notably, the anti-CCR5 monoclonal antibody T312 does not interfere with the binding of R5 HIV-1 to CCR5 [25]. Three subsets of resting (CD69^−^HLA-DR^−^) CD4^+^ T cells [T_N_ (CD45RA^+^CD27^+^CCR5^−^), CCR5^−^ T_M_ (CD45RA^−^CCR5^−^), and CCR5^+^ T_M_ (CD45RA^−^ CCR5^+^)] were isolated by flow-cytometric sorting using a FACSAria III (BD Biosciences, San Diego, CA, USA). Dead cells stained with the Live/Dead Fixable Violet Dead Cell Stain (ThermoFisher Scientific) were not isolated.

#### Infection and culture

Resting CD4^+^ T-cell subsets isolated by flow cytometry were infected with a 1:1 mixture of X4 and R5 viruses (determined by the amount of p24) at a range of 10–20 ng total p24 per 10^5^ cells by the spinoculation method as previously described [26]. After spinoculation, cells were washed and then incubated in R-10 medium [RPMI-1640 with 10% fetal bovine serum, 100 µg/ml penicillin, 100 µg/ml streptomycin, and 1% GlutaMAX solution (ThermoFisher Scientific)] for 2 h at 37 °C to induce viral fusion. Cells were then washed and cultured in R-10 medium supplemented with 1.25 µM saquinavir, to prevent viral spread, for up to 5 days. At 3 days post-infection, half of the culture medium was replaced with fresh medium, and the cultures were either kept in the resting condition or subjected to activation via the T-cell receptor using the Dynabeads Human T-Activator CD3/CD28 (ThermoFisher Scientific) in the presence of 5% heat-inactivated human AB serum and 50 U/ml IL-2 for 2 days. Flow cytometry was performed to detect productively infected cells at 3 days and 5 days post-infection using a FACSCanto II (BD Biosciences). All experiments with HIV-1 were conducted in a biosafety level 3 containment facility at National Institute of Infectious Diseases (NIID; Tokyo, Japan).

#### Analysis for flow cytometry

Data obtained from flow cytometry were saved as FCS files and analyzed using FlowJo v10.5.0 (BD Biosciences).

#### Statistical analysis

The significance of data comparisons was evaluated by repeated-measures one-way ANOVA and Tukey’s multiple comparison test using GraphPad Prism version 8 (Graph Pad Software, San Diego, CA, USA). A *P*-value of < 0.05 was considered statistically significant.

### Results

#### Profiles of resting T_N_-, CCR5^−^ T_M_-, and CCR5^+^ T_M_-cell populations prior to sorting

**Fig. 1:**
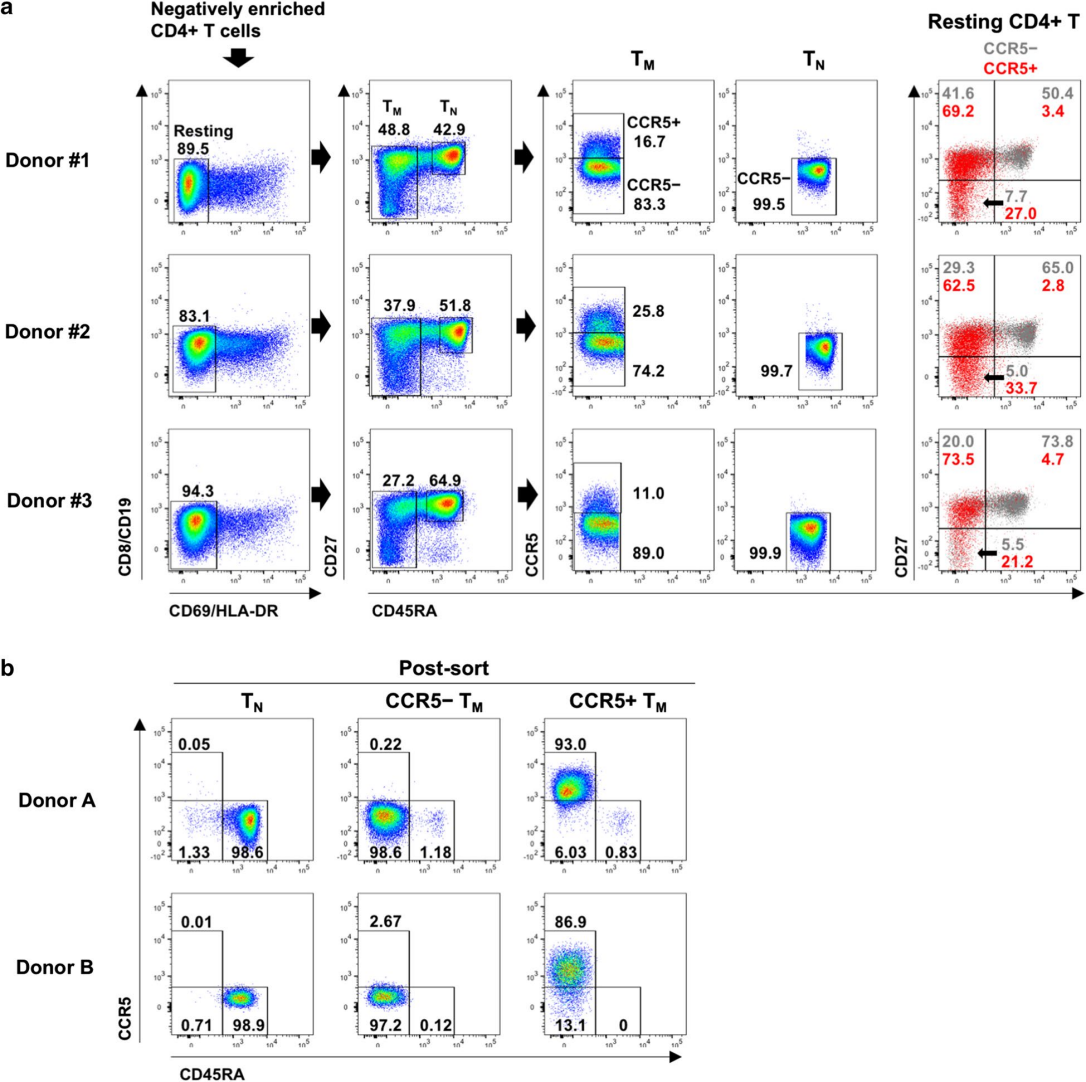
Flow-cytometric cell-sorting isolation strategy for three resting CD4^+^ T-cell subsets. **a** Pre-sorting flow-cytometry profiles of negatively enriched CD4^+^ T cells in PBMCs from three donors. Resting CD4^+^ T cells were negative for expression of CD8, CD19, CD69, and HLA-DR. Memory T cells (T_M_) were CD45RA^−^, whereas naïve T cells (T_N_) were CD45RA^+^CD27^+^. T_M_ cells were either CCR5^+^ or CCR5^−^, whereas T_N_ cells were CCR5^−^. The distribution of CCR5^+^ cells (red) and CCR5^−^ cells (gray) within resting CD4^+^ T cells according to expression of CD45RA and CD27 is shown in the right-hand set of panels. **b** Results of a pilot experiment to test the purity of three resting CD4^+^ T-cell subsets isolated by cell sorting using cell samples from two donors

For HIV-1 infection and culture experiments, negatively enriched CD4^+^ T cells were prepared from 200 ml each of peripheral blood from three donors. The enriched CD4^+^ T cells were stained with fluorochrome-conjugated antibodies and subjected to flow-cytometric sorting. Resting CD4^+^ T cells were defined as CD69 and HLA-DR double-negative, as described elsewhere [27], and represented 83.1–94.3% of the enriched CD4^+^ T cells (Fig. 1a). The resting CD4^+^ T cells consisted of CD45RA^+^CD27^+^CCR5^−^ T_N_, CD45RA^−^CCR5^−^ T_M_, and CD45RA^−^CCR5^+^ T_M_ cells, with CCR5 expressed exclusively on CD45RA^−^ T_M_ cells. The resting CCR5^+^ T_M_ cells were the minor population, with yields of 0.9 × 10^6^ to 2.2 × 10^6^ cells from the three donors. All of the sorted cells were used for HIV-1 infection and culture experiments. In a pilot experiment, the cell-sorting protocol was applied to small-scale samples from two more donors. Resting CCR5^+^ T_M_ cells were isolated at 86.9–93.0% purity, whereas resting T_N_ cells and CCR5^−^ T_M_ cells were isolated at > 97% purity (Fig. 1b).

#### Productive HIV-1 infection in resting cells

**Fig. 2:**
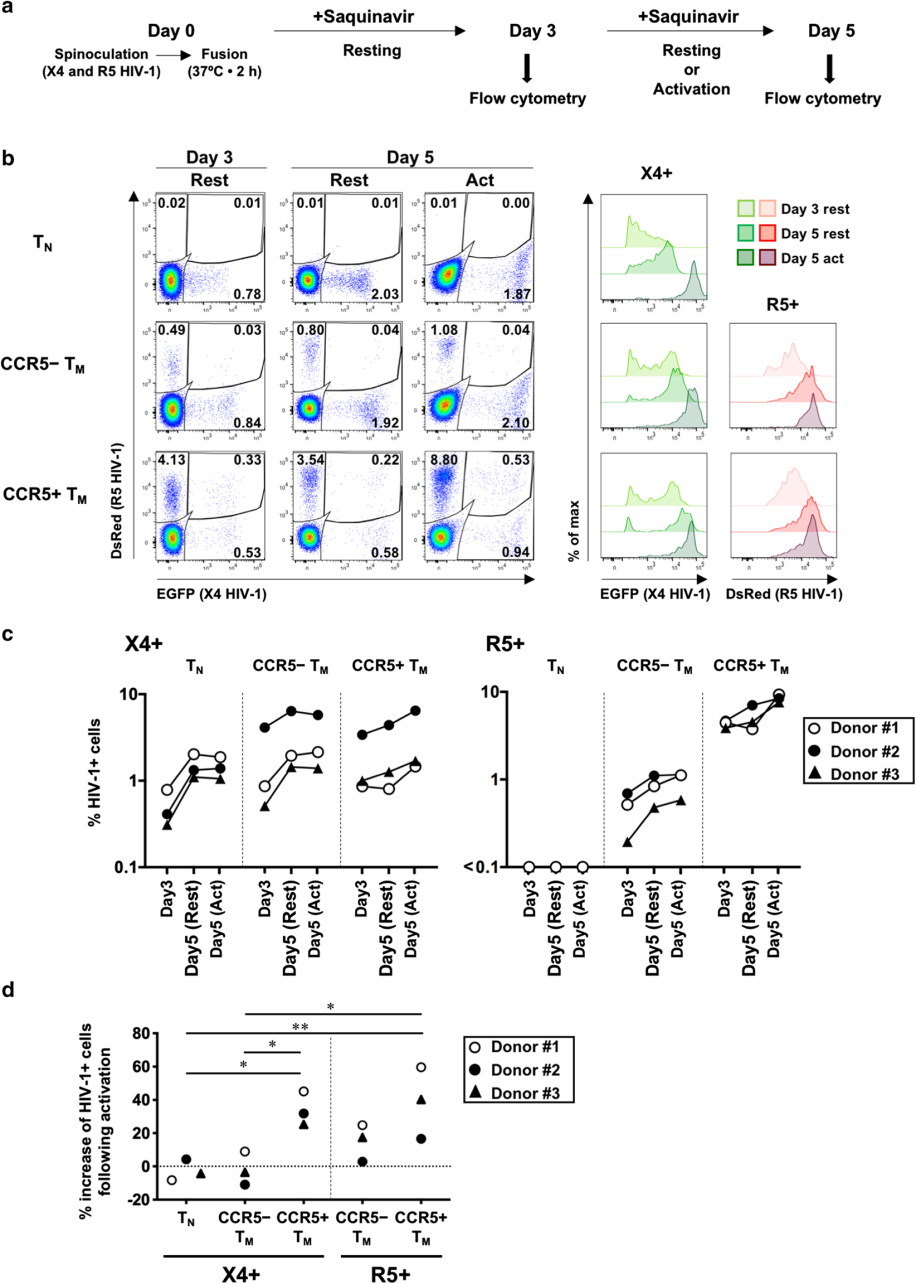
HIV-1 infection and culture of resting CD4^+^ T-cell subsets isolated by cell sorting. Subsets of naïve T cells (T_N_), or CCR5^+^ or CCR5^−^ memory T cells (T_M_), were separately infected and cultured. **a** Schematic of the protocol of HIV-1 infection and culture. **b** Representative flow-cytometry profiles of cells from Donor #1 at day 3 and day 5 post-infection (resting or activated), separated according to reporter expression indicating the presence of X4 or R5 HIV-1, with the percentage of each subset indicated (left panels). The intensity of fluorescence for each viral reporter in each cell subset [except for the very low percentage of DsRed^+^ cells (R5^+^) in T_N_ cells] is shown in the right-hand panels. **c** Percentages of HIV-1^+^ cells in each CD4^+^ T-cell subset in three donors. **d** Percentage increases in frequencies of HIV-1^+^ cells following activation were estimated by comparing percentages of HIV-1^+^ cells in the activation condition with those in the resting condition at day 5 post-infection. Significant differences (*P < 0.05, **P < 0.01) were determined by repeated-measures one-way ANOVA followed by Tukey’s multiple comparison test. In **c** and **d**, HIV-1^+^ cells include the corresponding reporter (either EGFP or DsRed) single-positive cells and double-positive cells

The protocol for the study of HIV-1 infection and culture is summarized in Fig. 2a. The results of flow cytometry showed that X4 HIV-1 productively infected all resting subsets, with EGFP expression in 0.50% ± 0.25% (mean ± SD) of T_N_ cells, 1.83% ± 1.99% of CCR5^−^ T_M_ cells, and 1.76% ± 1.43% of CCR5^+^ T_M_ cells at 3 days post-infection (Fig. 2b, c and Additional file 1). The percentage of resting cells with R5 HIV-1 infection was highest in CCR5^+^ T_M_ cells (4.35% ± 0.42%), considerably less in CCR5^−^ T_M_ cells (0.47% ± 0.25%), and very low in T_N_ cells (0.01% ± 0.01%) at 3 days post-infection. In T_N_ cells, extending the culture from 3 to 5 days in the resting condition substantially enhanced both the intensity and frequency of expression of the EGFP reporter, suggesting that productive infection in resting T_N_ cells proceeded more slowly than in resting T_M_ cells.

#### Activation increases the frequency of HIV-1 infection in CCR5^+^ T_M_ cells

Proportions of HIV-1^+^ cells 5 days post-infection were compared in resting cells and cells that were activated on day 3 post-infection, to assess the presence of transcription-silent, replication-competent viral reservoirs (Fig. 2d). Notably, in the CCR5^+^ T_M_-cell subset, the proportions of both R5 HIV-1^+^ cells and X4 HIV-1^+^ cells were higher in activated cells than in resting cells. In the CCR5^−^ T_M_-cell subset, the frequency of R5 HIV-1^+^ cells was higher following activation, but the difference was not significant. No consistent response to activation in terms of the frequency of X4 HIV-1^+^ cells was observed in either T_N_ or CCR5^−^ T_M_ subset, despite that the robust activation was observed on the basis of the reporter intensity (Fig. 2b and Additional file 1).

### Discussion

In this study, we demonstrated by flow cytometry that resting CD4^+^ T cells are subject to productive infection. This result is consistent with findings from other studies, where primary resting CD4^+^ T cells were infected with HIV-1 expressing fluorescent reporter proteins [22, 28, 29]. The profile of productive infection that we observed was associated with the expression pattern of the coreceptors. In particular, CCR5 is expressed on T_M_ cells, especially the T_EM_ subset, and is rarely detectable on T_N_ cells, whereas CXCR4 is detectable in all CD4^+^ T-cell subsets [30, 31]. The R5 HIV-1^+^ cells that we detected in the CCR5^−^ T_M_ subset might result from the presence of R5 HIV-1-permissive cells that were not detected for CCR5 expression by flow cytometry, as low levels of CCR5 are sufficient for R5 HIV-1 infection if they are associated with sufficient cell-surface expression of CD4 [32].

We found that the intensity of the viral reporter fluorescence in resting CD4^+^ T_N_ cells was lower than in resting CD4^+^ T_M_ cells at 3 days post-infection. Previous studies indicated that the low intensity of the viral reporter fluorescence may suggest the presence of unintegrated viral DNA, which is capable of generating infectious virions [28, 29]. Although we did not determine the levels of integrated proviral DNA and unintegrated viral DNA, our results highlight a replicative advantage of CCR5^+^ memory subsets for both X4 and R5 HIV-1, even in the resting condition.

Our main aim was to determine which subsets of resting CD4^+^ T cells contribute to formation of the latent reservoir in the presence of both X4 and R5 HIV-1. When we compared activated and resting cells at day 5 post-infection, we found consistently higher proportions of R5 HIV-1^+^ cells and X4 HIV-1^+^ cells in the CCR5^+^ T_M_-cell subset following activation. This effect of activation was not seen in T_N_ or CCR5^−^ T_M_ cells. We previously showed that resting CD4^+^ T_N_ cells resist latent HIV-1 reactivation [33], supporting the present result. Latent HIV-1 reservoirs may exist in T_N_ and CCR5^−^ T_M_ cells, as it is possible that the level of depletion by viral cytopathy just exceeded or was equal to that of generation from latency. However, our results suggest that resting CCR5^+^ T_M_ cells are the major reservoir of replication-competent, latent R5 HIV-1 and X4 HIV-1 among resting CD4^+^ T-cell subsets.

## Limitations

First, we did not investigate whether, after exposure to virus, reporter-negative resting cells possessed proviruses that were induced for viral replication following activation. Second, because we identified preferential infection of R5 HIV-1 over X4 HIV-1 in resting CCR5^+^ T_M_ cells, it was assumed that R5 HIV-1 is more permissive for latent infection in resting CCR5^+^ T_M_ cells than X4 HIV-1. However, it has been reported that X4 laboratory strains such as NL4-3, from which our X4 HIV-1 (HIV-1_NL-E_) was derived, are less effective at infecting cells expressing low levels of CXCR4 than primary isolates [34]. Indeed, CXCR4 expression levels in CCR5^+^ T_M_ cells are lower than in T_N_ cells (Additional file 2) [31]. Our separate experiments demonstrated that the predominance of R5 HIV-1 over X4 HIV-1 had already begun at the binding stage of the infection of CCR5^+^ T_M_ cells (Additional file 3). Therefore, X4 primary isolates should be tested to evaluate the predominance of latent infection between X4 and R5 viruses.

## Additional files

**Additional file 1: Fig. S1.** Flow-cytometry profiles of Donor #2 and Donor #3 at day 3 and day 5 post-infection.

**Additional file 2: Fig. S2.** Comparison of CXCR4 expression between T_N_ and CCR5^+^ T_M_ cells within resting CD4^+^ T cells.

**Additional file 3: Fig. S3.** Data from separate experiments for the evaluation of HIV-1 binding (**S3-1**), entry (**S3-2**), and reverse transcription (**S3-3**) in CCR5^+^ T_M_ cells.

## Abbreviations

NIID: National Institute of Infectious Diseases
PBMC: peripheral blood mononuclear cell
R5: CCR5-tropic
T_CM_ cell: central memory T cell
T_EM_ cell: effector memory T cell
T_M_ cell: memory T cell
T_N_ cell: naïve T cell
T_TM_ cell: transitional memory T cells
X4: CXCR4-tropic
