# Correction to: A CCR5^+^ memory subset within HIV-1-infected primary resting CD4^+^ T cells is permissive for replication-competent, latently infected viruses in vitro

**DOI:** 10.1186/s13104-019-4357-2

**Published:** 2019-06-10

**Authors:** Kazutaka Terahara, Ryutaro Iwabuchi, Masahito Hosokawa, Yohei Nishikawa, Haruko Takeyama, Yoshimasa Takahashi, Yasuko Tsunetsugu-Yokota

**Affiliations:** 1 Department of Immunology, National Institute of Infectious Diseases, 1-23-1 Toyama, Shinjuku-ku, Tokyo, 162-8640 Japan; 2 Department of Life Science and Medical Bioscience, Waseda University, 2-2 Wakamatsu-cho, Shinjuku-ku, Tokyo, 162-8480 Japan; 3 Computational Bio Big-Data Open Innovation Laboratory, National Institute of Advanced Industrial Science and Technology, 3-4-1 Okubo, Shinjuku-ku, Tokyo, 169-8555 Japan; 4 Research Organization for Nano & Life Innovation, Waseda University, 513 Wasedatsurumaki-cho, Shinjuku-ku, Tokyo, 162-0041 Japan; 5 Institute for Advanced Research of Biosystem Dynamics, Waseda Research Institute for Science and Engineering, Waseda University, 2-2 Wakamatsu-cho, Shinjuku-ku, Tokyo, 162-8480 Japan; 6 Department of Medical Technology, School of Human Sciences, Tokyo University of Technology, 5-23-22 Nishikamata, Ota-ku, Tokyo, 144-8535 Japan

## Correction to: BMC Res Notes (2019) 12:242 10.1186/s13104-019-4281-5

After publication of the original article [1], the authors became aware of a miscalculation in the original Fig. 2d.

$$ \frac{{\% \,{\text{HIV-}}1^{ + \,} {\text{activated}}\,{\text{cells}}\,{\text{at}}\,{\text{Day}}\,5 - \% \,{\text{HIV-}}1^{ + \,} \,{\text{resting}}\,{\text{cells}}\,{\text{at}}\,{\text{Day}}\,5}}{{\% \,{\text{HIV-}}1^{ + \,} {\text{activated}}\,{\text{cells}}\,{\text{at}}\,{\text{Day}}\,5}} \times 100 $$

should be calculated as:

$$ \frac{{\% \,{\text{HIV-}}1^{ + \,} {\text{activated}}\,{\text{cells}}\,{\text{at}}\,{\text{Day}}\,5 - \% \,{\text{HIV-}}1^{ + \,} \,{\text{resting}}\,{\text{cells}}\,{\text{at}}\,{\text{Day}}\,5}}{{\% \,{\text{HIV-}}1^{ + \,} \,{\text{resting}}\,{\text{cells}}\,{\text{at}}\,{\text{Day}}\,5}} \times 100 $$

The corrected Fig. 2d is shown in this erratum.

**Fig. 2:**
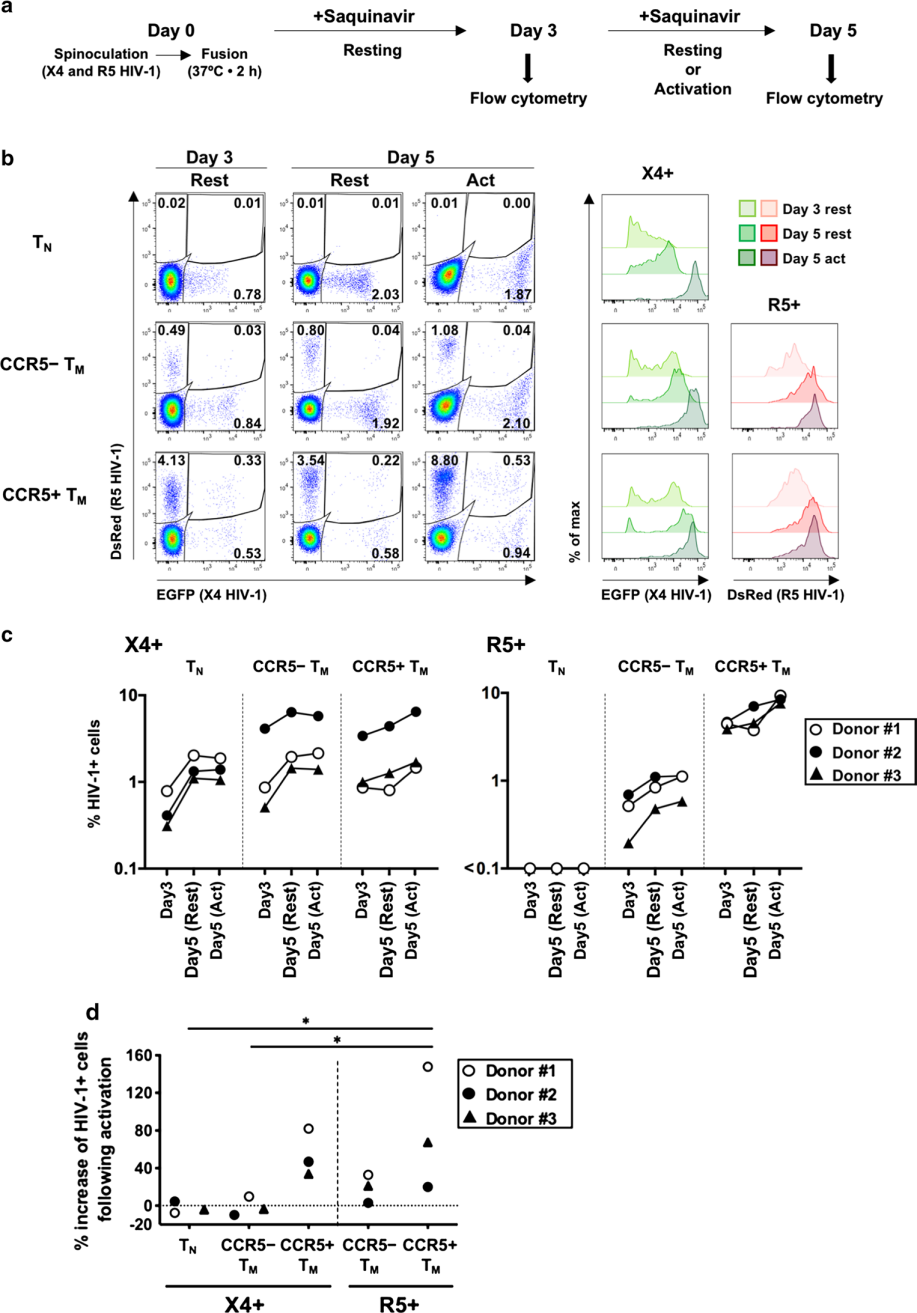
HIV-1 infection and culture of resting CD4^+^ T-cell subsets isolated by cell sorting. Subsets of naïve T cells (T_N_), or CCR5^+^ or CCR5^−^ memory T cells (T_M_), were separately infected and cultured. **a** Schematic of the protocol of HIV-1 infection and culture. **b** Representative flow-cytometry profiles of cells from Donor #1 at day 3 and day 5 post-infection (resting or activated), separated according to reporter expression indicating the presence of X4 or R5 HIV-1, with the percentage of each subset indicated (left panels). The intensity of fluorescence for each viral reporter in each cell subset [except for the very low percentage of DsRed^+^ cells (R5^+^) in T_N_ cells] is shown in the right-hand panels. **c** Percentages of HIV-1^+^ cells in each CD4^+^ T-cell subset in three donors. **d** Percentage increases in frequencies of HIV-1^+^ cells following activation were estimated by comparing percentages of HIV-1^+^ cells in the activation condition with those in the resting condition at day 5 post-infection. Significant differences (*P < 0.05, **P < 0.01) were determined by repeated-measures one-way ANOVA followed by Tukey’s multiple comparison test. In **c** and **d**, HIV-1^+^ cells include the corresponding reporter (either EGFP or DsRed) single-positive cells and double-positive cells

Although the statistical significances have been altered, the hierarchical mode between cell-subset groups remains the same. It is still shown that numbers of X4 HIV-1^+^ cells increased consistently in the CCR5^+^ TM subset of all three donors tested. Therefore, the correction does not change the scientific conclusion.
