# Supplementary figures and images for: A CCR5+ memory subset within HIV-1-infected primary resting CD4+ T cells is permissive for replication-competent, latently infected viruses in vitro
Source: BMC Res Notes. 2019 Apr 29;12:242. doi: 10.1186/s13104-019-4281-5 (PMC6489248; doi:10.1186/s13104-019-4281-5)

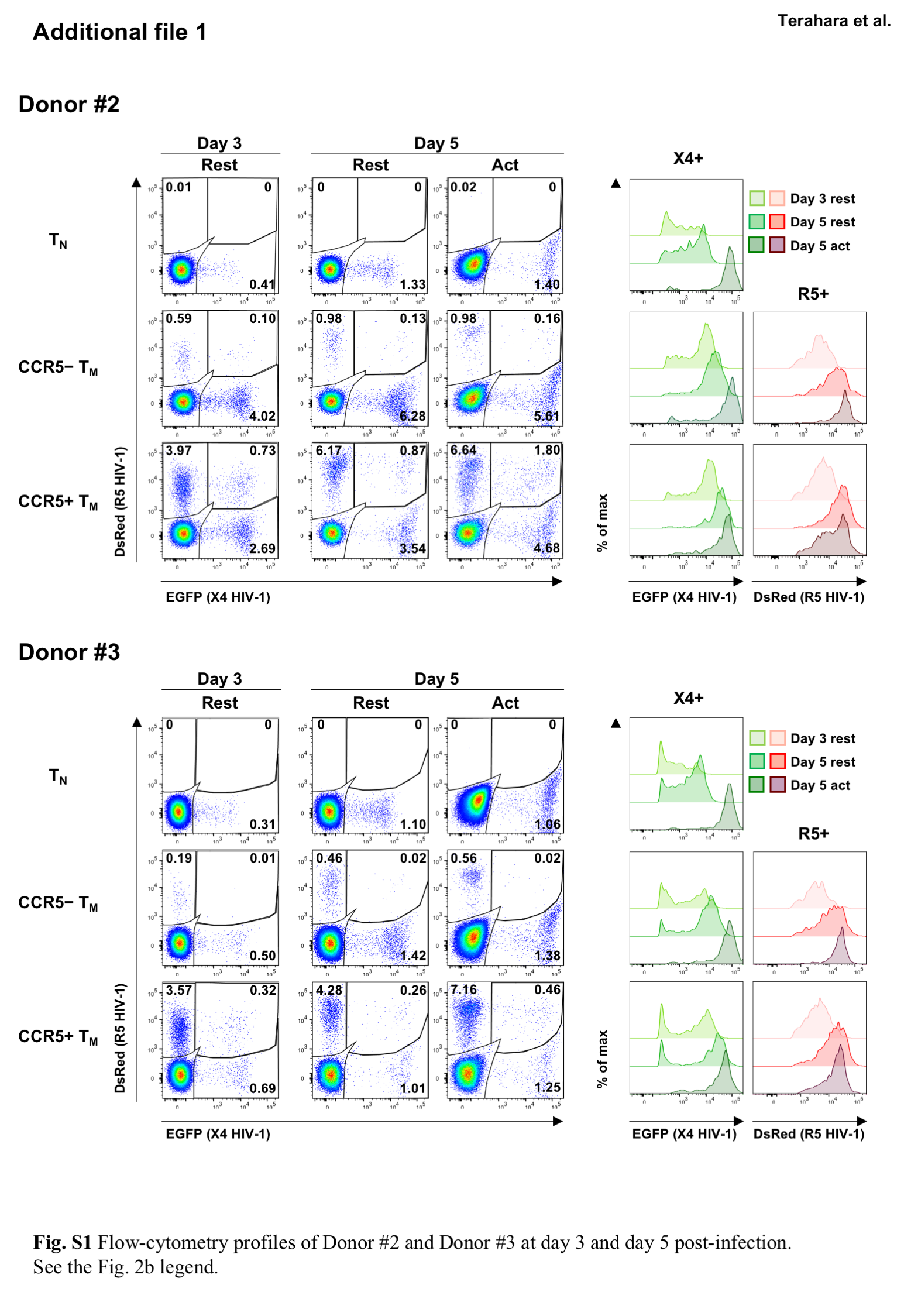

Supplement: Supplementary file 1 — Additional file 1: Fig. S1. Flow-cytometry profiles of Donor #2 and Donor #3 at day 3 and day 5 post-infection. [file 13104_2019_4281_MOESM1_ESM.tiff]

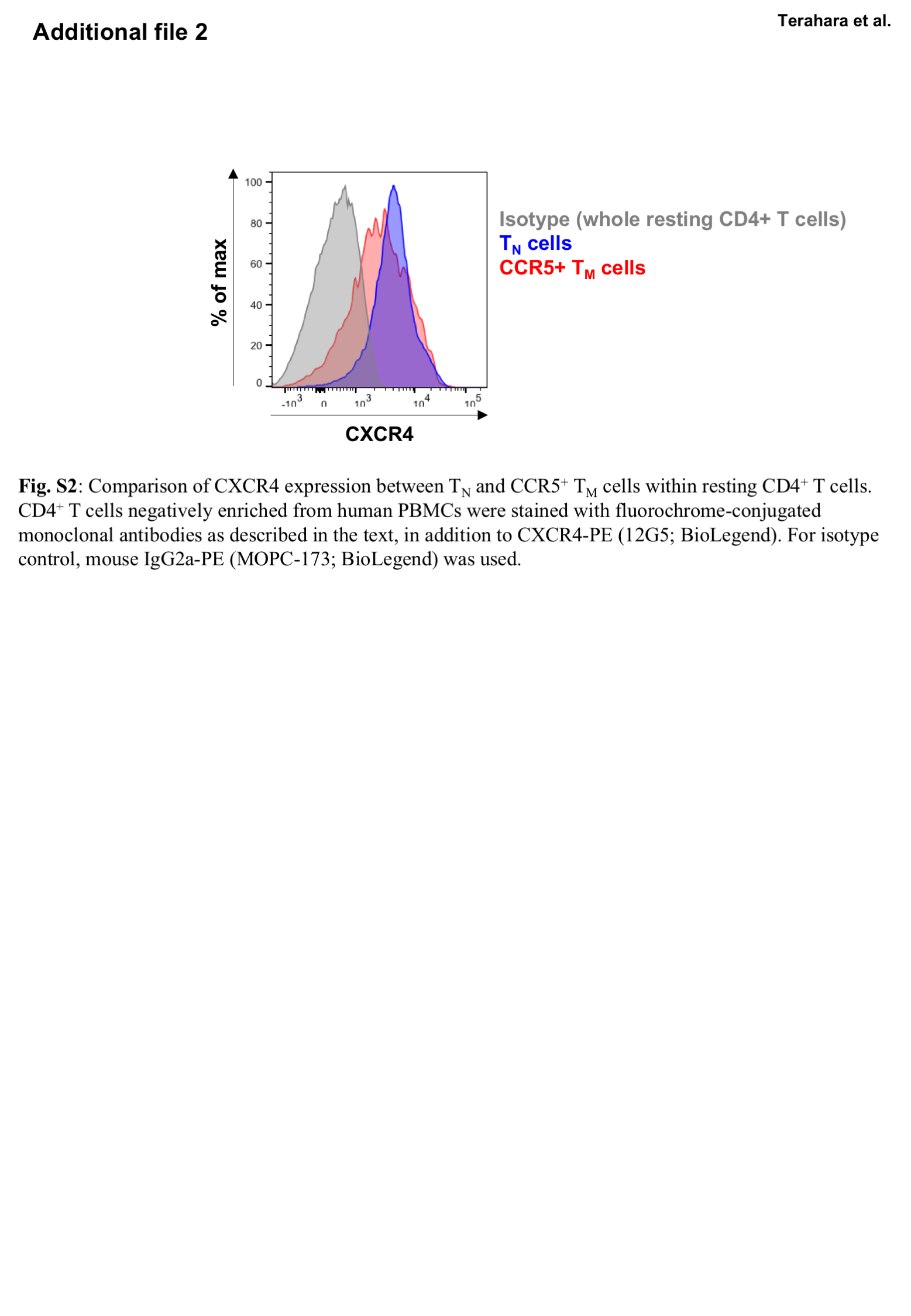

Supplement: Supplementary file 2 — Additional file 2: Fig. S2. Comparison of CXCR4 expression between TN and CCR5+ TM cells within resting CD4+ T cells. [file 13104_2019_4281_MOESM2_ESM.tiff]
