# Supplementary material for: A CCR5+ memory subset within HIV-1-infected primary resting CD4+ T cells is permissive for replication-competent, latently infected viruses in vitro
Source: BMC Res Notes. 2019 Apr 29;12:242. doi: 10.1186/s13104-019-4281-5 (PMC6489248; doi:10.1186/s13104-019-4281-5)
Supplement: Supplementary file 3 — Additional file 3: Fig. S3. Data from separate experiments for the evaluation of HIV-1 binding (S3-1), entry (S3-2), and reverse transcription (S3-3) in CCR5+ TM cells. [file 13104_2019_4281_MOESM3_ESM.docx]

**Additional file 3: Data from separate experiments for the evaluation of HIV-1 binding (Fig. S3-1), entry (Fig. S3-2), and reverse-transcription (Fig. S3-3) in CCR5^+^ T_M_ cells**

**Fig. S3-1: Assay for HIV-1 binding**

Sort-isolated resting CCR5^+^ T_M_ cells were separately infected with X4 and R5 viruses, in each case with the same amount of p24 (20 ng per 10^5^ cells), by spinoculation. Cells were then washed, and viral and cellular RNA was extracted using the NucleoSpin RNA XS (Macherey-Nagel, Düren, Germany). Extracted RNA was subjected to qRT-PCR targeting the *gag* region, as previously described [1]. RNase P mRNA as an internal control was also quantitated, as previously described [2]. Normalized copy numbers of bound HIV-1 are shown. As a result, R5 HIV-1 bound to resting CCR5^+^ T_M_ cells 2.2-fold higher copy number than X4 HIV-1 on average (*n* = 5). A significant difference (*P* = 0.0396) was determined by the paired *t*-test.


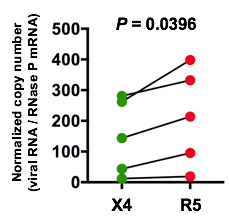


**Fig. S3-2: Assay for HIV-1 entry**

For HIV-1 entry, a fusion assay was performed as previously described [1]. Sort-isolated resting CCR5^+^ T_M_ cells were infected by spinoculation with a mixture of X4 and R5 viruses (either of which possessed β-lactamase-Vpr chimeric proteins), with the same amount of p24 for each, at 20 ng per 10^5^ cells. Cells permissive for HIV-1 fusion were detected using the GeneBLAzer *In Vivo* Detection Kit (ThermoFisher Scientific) by flow cytometry. As a result, the frequency of R5 HIV-1-fused cells was 1.5-fold higher than that of X4 HIV-1-fused cells on average (*n* = 4). A significant difference (*P* = 0.0157) was determined by the paired *t*-test.


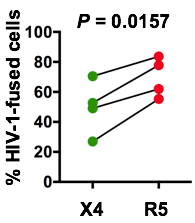


**Fig. S3-3: Assay for HIV-1 reverse transcription (RT) products**

For HIV-1 RT products, sort-isolated resting CCR5^+^ T_M_ cells were infected with a mixture of X4 and R5 viruses with the same amount of p24 for each (20 ng per10^5^ cells) by spinoculation. At 22 h post-infection, cells cultivated in the resting condition were washed and lysed in the presence of 0.6% Nonidet P-40. A 500-cell-equivalent aliquot was subjected to qPCR as previously described [3], targeting EGFP and DsRed DNA with the following primers and probes; EGFP forward primer: 5’-ACGTAAACGGCCACAAGTTC-3’, EGFP reverse primer: 5’-AAGTCGTGCTGCTTCATGTG-3’, EGFP TaqMan probe: 5’-FAM-TGCAGTGCTTCAGCCGCTAC-MGBNFQ-3’, DsRed forward primer: 5’-cggctccttcatctacaagg-3’, DsRed reverse primer: 5’-GGTGATGTCCAGCTTGGAGT-3’, and DsRed TaqMan probe: 5’-VIC-tacatggccaagaagcccgt-MGBNFQ-3’. The copies per cell of RT products of R5 HIV-1 was higher than that of X4 HIV-1 in all three donors tested, although the difference was not significant by the paired *t*-test (*P* = 0.0903).


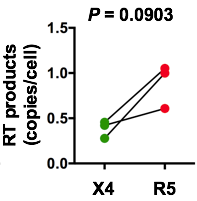


**References**

1. Terahara K, Ishige M, Ikeno S, Mitsuki YY, Okada S, Kobayashi K, Tsunetsugu-Yokota Y. Expansion of activated memory CD4+ T cells affects infectivity of CCR5-tropic HIV-1 in humanized NOD/SCID/JAK3null mice. PLOS ONE. 2013;8:e53495.

2. Ikeno S, Suzuki MO, Muhsen M, Ishige M, Kobayashi-Ishihara M, Ohno S, Takeda M, Nakayama T, Morikawa Y, Terahara K, et al. Sensitive detection of measles virus infection in the blood and tissues of humanized mouse by one-step quantitative RT-PCR. Front Microbiol. 2013;4:298.

3. Yoda T, Hosokawa M, Takahashi K, Sakanashi C, Takeyama H, Kambara H. Site-specific gene expression analysis using an automated tissue micro-dissection punching system. Sci Rep. 2017;7:4325.
